# Selectivity of 1-*O*-Propargyl-d-Mannose Preparations

**DOI:** 10.3390/molecules27051483

**Published:** 2022-02-22

**Authors:** Ilona Krabicová, Bohumil Dolenský, Michal Řezanka

**Affiliations:** 1 Department of Chemistry, Faculty of Science, Humanities and Education, Technical University of Liberec, Studentská 1402/2, 46117 Liberec, Czech Republic; ilona.krabicova@tul.cz; 2 Department of Analytical Chemistry, Faculty of Chemical Engineering, University of Chemistry and Technology Prague, Technická 5, 16628 Prague, Czech Republic; bohumil.dolensky@vscht.cz; 3 Department of Nanochemistry, Institute for Nanomaterials, Advanced Technologies and Innovation, Technical University of Liberec, Studentská 1402/2, 46117 Liberec, Czech Republic

**Keywords:** alkylation, furanose, NMR spectroscopy, mannose, propargyl, pyranose

## Abstract

Thanks to their ability to bind to specific biological receptors, mannosylated structures are examined in biomedical applications. One of the most common ways of linking a functional moiety to a structure is to use an azide-alkyne click reaction. Therefore, it is necessary to prepare and isolate a propargylated mannose derivative of high purity to maintain its bioactivity. Three known preparations of propargyl-α-mannopyranoside were revisited, and products were analysed by NMR spectroscopy. The preparations were shown to yield by-products that have not been described in the literature yet. Our experiments showed that one-step procedures could not provide pure propargyl-α-mannopyranoside, while a three-step procedure yielded the desired compound of high purity.

## 1. Introduction

Mannose is a natural monosaccharide, occurring in plants as well as microorganisms, usually as a constituent of mannan, hemicellulose, or cellulose [1]. Historically, mannose was used in the food industry as an alternative sweetener or to improve the texture of foodstuff, in cosmetics, or it was used as a feed additive [1]. Thanks to its ability to bind to specific biological receptors, mannose has been examined as a functional moiety of biosensors [2,3,4] and as the key compound for bacterial labels [5]. However, there is no more discussed application of mannose than its use in medicine. The number of articles published on mannosylated structures increases every year, and a frequent theme is the preparation of mannosylated nanocarriers capable of targeted delivery [6,7,8,9,10] or cell imaging [11,12]. Several types of cells, including antigen-presenting cells, dendritic cells, macrophages, and several tumorous cells, are able to express mannose receptors as an important component of the immune system [6,13]. The main function of these transmembrane proteins is to bind mannosylated pathogens and glycoproteins in the organism, thereby facilitating phagocytosis [13]. Therefore, functionalisation of nanostructures with mannose may enhance targeting into a specific tissue, as well as enhancing the cellular uptake of drugs incorporated into a carrier.

For the preparation of a mannose-functionalised material, the “click” alkyne–azide cycloaddition reaction is frequently used [14,15,16]. To perform this reaction, the synthesis of the alkyne derivative of mannose is essential.

As mentioned before, mannose derivatives have become a point of interest for possible application in targeted drug delivery. However, organisms are very sensitive to the right form of the mannose derivative structure. Mannose contains six carbons and appears in either a pyranose or furanose form [1]. Therefore, the synthesis of targeting nanocarriers requires high selectivity and exact reproducibility of the products. In an aqueous solution, d-mannose appears in the α-d-pyranose form (approximately 68%) and the β-d-pyranose form (32%) at room temperature [17]. The carbohydrate-recognition domains of mannose receptors contain mannose-binding lectin, which recognises the equatorial C3 and C4 hydroxyl groups on the pyranose ring [18]. This fact shows that it is crucial to deeply examine the synthesis product and properly separate the undesired by-products such as the furanose form.

**Scheme 1:**
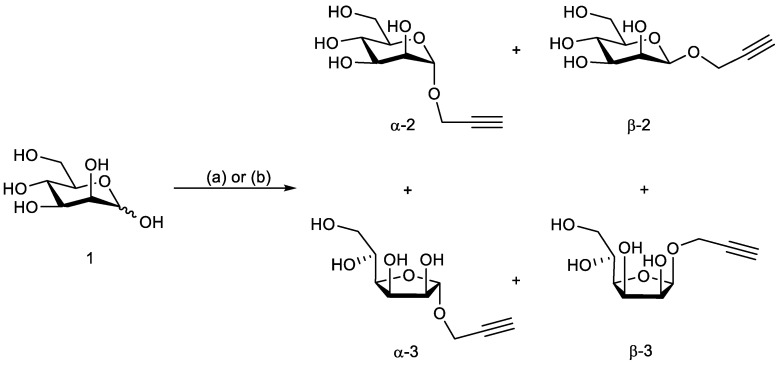
One-step procedure for the preparation of propargyl-mannosides. The synthesis was performed according to two different methods: (**a**) propargyl alcohol, HCl, RT, overnight [22]; (**b**) propargyl alcohol, H_2_SO_4_ on silica, 65 °C, 2 h [19].

Propargyl-mannosides are synthesised using two different approaches—direct synthesis (Scheme 1, conditions (a)) and three-step synthesis using the protection–deprotection strategy. Several authors mentioned that the preparation they used may lead to the desired mannopyranoside but that they also may lead to unknown by-products [19,20,21]. Despite that, some authors used the product immediately without purification and estimation of purity [22,23]. For example, Richards et al. [22] used direct synthesis catalysed by hydrochloric acid for the propargylation of mannose and galactose. The products of the preparation were used without any further purification, no yields were given, and the NMR characteristics were insufficient.

Other preparations were made with sulphuric acid as a catalyst (Scheme 1, conditions (b)) [16,19,20,21,24,25]. All authors purified the product using column chromatography, although the mobile phases differed (CHCl_3_–MeOH; EtOAc–acetone–H_2_O; CH_2_Cl_2_–MeOH). An overview of the results is given in Table 1. The yields of the propargylation vary (30–90%) as well as the anomeric purity of the products (the ratio of α/β mannopyranoside differs from 1:0 to 2:1). Although Roy et al. [19] mentioned that furanosides may also form, they were not described.

In addition to the strategies examined in this article, the *O*-propargylation of mannose can be performed, e.g., via trichloroacetimidate intermediate product [26,27,28,29], or a thio-derivative intermediate [30,31]. However, these approaches may also result in the mixture of α- and β-d-mannopyranoside [27,30].

The mannose propargylation products formed by various synthetic procedures are the main subject of this article. Structures were examined by NMR, and the spectra are shown to provide the reader with the characterisation data of all forms, thereby facilitating the future synthesis and identification of 1-*O*-propargyl-mannosides.

## 2. Results and Discussion

In our work, the one-step approach was repeated according to Richards et al. [22] to a total yield of 25%. However, not only propargyl-α-d-mannopyranoside (α-2) was detected using NMR (see below) but also other by-products in the form of other isomers were detected, such as propargyl-β-d-mannopyranoside (β-2), propargyl-α-mannofuranoside (α-3), and propargyl-β-mannofuranoside (β-3) in the ratio of 28:12:51:9. The calculated yields (based on mannose) were 7% α-2, 3% β-2, 13% α-3, and 2% β-3. Unfortunately, not even thorough purification by column chromatography succeeded in the perfect separation of the products. The procedure was repeated several times with similar results. The isomers were not reported by Richards et al. [22], who used the product for further synthesis without any purification.

We recognised furanosides **3** and pyranosides **2** based on the ^1^H-^13^C HMBC NMR spectra. The spectra of furanosides **3** revealed a strong interaction between H1–C4 (^3^J_HC_), and a negligible interaction between H1–C5 (^4^J_HC_), while pyranosides **2** had a negligible H1–C4 (^4^J_HC_), and strong H1–C5 (^3^J_HC_) interaction. The anomers were identified based on NOESY and HSQC-NOESY NMR spectra, where H1 had NOE only to H2 and propargyl CH_2_ in the case of α-anomers, but also to H3, H4, or H5 in the case of β-anomers. When the NMR samples in D_2_O were acidic (pH ~ 5), then the acetylene hydrogen was exchanged during a few days by deuterium, which was well recognised as the multiplicity of propargyl CH_2_ hydrogen signals decreased while the multiplicity of acetylene carbon signals increased. In addition, the ^1^H/^2^H isotope effect on their chemical shifts was observed (see the Supplementary Materials, Table S1).

The sulfuric acid-catalysed preparation (Scheme 1, conditions (b)) is used most often. We revisited the sulfuric acid-catalysed preparation [19] to compare the effect of different reaction conditions on direct synthesis. The yields reported in the literature vary widely from 30% to 90% (Table 1). Our results lie within this interval, as we obtained a 37% yield. However, only a minority of authors described the synthesis of any product other than propargyl-α-d-mannopyranoside (α-2), specifically propargyl-β-d-mannopyranoside (β-2) was detected in a few cases [20,21]. Moreover, the products obtained from the reaction were commonly per-*O*-acetylated before characterisation. In our work, non-acetylated products were investigated, and both pyranosides α-2 and β-2 and furanosides α-3 and β-3 were detected in molar ratios of 712:209:57:22, respectively. The calculated yields (based on mannose) were 26% α-2, 8% β-2, 2% α-3, and 1% β-3. To the best of our knowledge, mannofuranosides produced by this reaction have never been described.

**Scheme 2:**
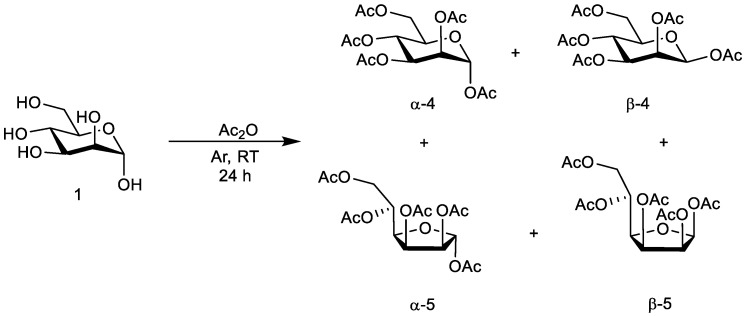
The first step of the three-step synthesis using protection via acetylation.

Next, the products of the three-step propargyl-mannoside preparation were also examined. In the first step (Scheme 2), per-*O*-acetylated mannopyranoside was obtained with the majority of per-*O*-acetyl-α-d-mannopyranoside. Our detailed analysis of the ^1^H NMR spectrum of the product revealed the presence of both pyranoside anomers and traces of furanoside anomers. In the literature, the formation of the mixture of both pyranosides is described, but not quantified (Table 2). However, the anomeric ratio of α- and β-pyranoside in the product was reported in the articles, where a different synthetic procedure was used with the same aim (specifically α/β-pyranoside in the ratio of 4.75:1) [32,33]. No furanosides **3** were reported. During the characterisation of our products, the major anomer α-4 was well identified based on the medium NOE of H1 to acetyl on C2, and weak to acetyl on C3, which was missing in the case of anomer β-4. Further NMR evidence of the configuration and conformation of α-4 was prevented due to overlaps and second-order character of the signals. In contrast, the minor anomer β-4 had well-dispersed ^1^H signals. The observation of strong NOE of H1 on signals of the H2, H3, and H5 proton and weak to acetyl on C1 only proves the configuration. The pyranose ring conformation of β-4 shown Scheme 2 is in accordance with the gain of ^1^H–^1^H interaction constants. In addition, based on selective 1D TOCSY NMR experiments, we identified the presence of peracetylated α-d-mannofuranoside α-5 and β-d-mannofuranoside β-5. The obtained characteristics were in accordance with the published data [34]. (We probably recognized a typo in the article; the chemical shift of H3 of β-d-mannofuranoside should read 5.651 ppm.) Mannosides α-4, β-4, α-5, and β-5 were in the molar ratio of 860:129:10:1, which corresponds to yields of 66%, 10%, 1%, and 0.1%, respectively. We repeated this experiment with identical results.

**Scheme 3:**
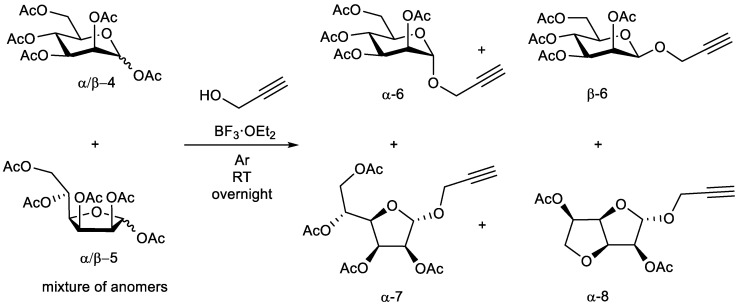
The second step of the three-step synthesis using protection via acetylation.

In the second step (Scheme 3), the mixture of α-4, β-4, α-5, and β-5 was used as the starting material, as it was not possible to separate the individual compounds. In contrast to the literature, which refers to only one product in the form of α-pyranoside (Table 3), the reaction resulted in another mixture of products, including the desired α-6, but also β-6, propargyl-tetraacetyl-α-furanose (α-7), and propargyl-anhydromannose (α-8). NMR analysis revealed the anomers were in the molar ratio of 9803:149:32:7, which corresponds to the yields of 82% for α-6 (based on α-4), 8% for β-6 (based on β-4), 23% for α-7 (based on α-5), and 5% for α-8 (based on α-5). However, during the purification by column chromatography, it was possible to separate the mixture into three fractions, including a fraction of pure α-6, which was used for the next step of the synthesis. The compound α-8 was identified by correlation 2D NMR experiments; the configuration was suggested based on NOE experiments and similarity with the known analogues [38]. Several ^1^H NMR signals were overlapped, so we also recorded the NMR spectra in C_6_D_6_, in which the signals were more separated, and the NOE spectra were more reliable. Fortunately, we also isolated a fraction with a high content of β-6; therefore, the comparison of NOE unambiguously confirmed the configuration on carbon C1 (see the Supplementary Materials, Table S1).

**Scheme 4:**
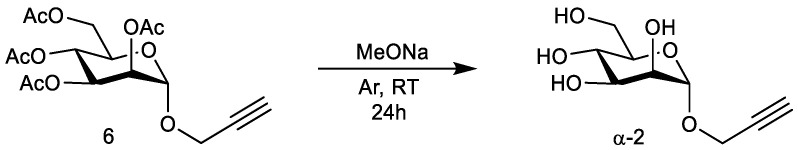
The last step of the three-step synthesis using protection via acetylation.

The last step (Scheme 4) based on the deprotection of the hydroxyl groups of mannose [39,43] resulted in the production of pure propargyl-α-d-mannopyranoside (α-2), which is in agreement with the literature, which describes only the α-pyranoside form in 89–98% [32,33,35,39,41,44,45]. The NMR characteristics of all products are listed in the Supplementary Materials, Table S1.

## 3. Materials and Methods

### 3.1. Materials

Synthetic d-(+)-mannose was purchased from Sigma-Aldrich, along with propargyl alcohol and acetic anhydride. Hydrochloric acid, sulphuric acid, ethyl acetate (EtOAc), and dichloromethane (DCM) were acquired from Lachner; methanol (MeOH) and pyridine were acquired from Penta. Boron trifluoride diethyl etherate (BF_3_⋅OEt_2_) was purchased from TCI. Anhydrous DCM was obtained from Acros. Methanol was further dried over molecular sieves (4Å pellets, Acros), and DCM was distilled before using to eliminate impurities. Other chemicals were used as supplied. Sodium methoxide was prepared according to the standard procedure by the reaction of sodium (2.3 g) with anhydrous methanol (100 mL) in an argon atmosphere.

### 3.2. Instruments

The NMR spectra were recorded by a 500 MHz instrument (JEOL, Tokyo, Japan) at 25 °C. The chemical shifts (δ) are presented in ppm, and the coupling constants (*J*) are presented in Hz. The ^1^H and ^13^C chemical shifts are referenced to tetramethylsilane using the solvent signals HDO 4.66 ppm, CHCl_3_ 7.26 ppm, CHD_2_OD 3.31 ppm, and C_6_HD_5_ 7.16 ppm for ^1^H and CDCl_3_ 77.16 ppm, CD_3_OD 49.00 ppm, and C_6_D_6_ 128.06 for ^13^C. For the estimation of molecular structures, a series of standard NMR experiments was recorded and analysed, including 2D COSY, HSQC, HMBC, TOCSY, NOESY, ROESY, and selective 1D NOESY, 1D TOCSY, and selective homodecoupled ^1^H NMR spectra. For the mass spectrometry (MS) analysis, a Sciex X500R QTOF HR mass spectrometer was used. Fourier-transform infrared analysis (FTIR) was performed using a Nicolet iZ10 from ThermoFisher Scientific (Waltham, MA, USA). The observed pseudo molecular peak [M + Na]^+^ of the studied compounds was usually followed by a less intensive peak of [2M + Na]^+^, which is typical for oxygen compounds [46]. The peak of [3M + Na]^+^ was not observed. A detailed anomeric composition of the products is given in the Results and Discussion section.

### 3.3. Direct Synthesis

#### 3.3.1. Synthesis of 1-*O*-Propargyl-d-Mannose (**2**) Catalysed by Hydrochloric Acid

Direct synthesis catalysed by hydrochloric acid was performed according to the procedure described by Richards et al. [22]. Briefly, mannose (300 mg, 1.67 mmol) and propargyl alcohol (3 mL, 86.9 mmol) were stirred in a round bottom flask. Subsequently, hydrochloric acid (0.15 mL, 0.45 mmol) was added, and the mixture was stirred overnight. The solution was concentrated in vacuo, and the products were purified by column chromatography (DCM/MeOH 9:1), giving two fractions: fraction A (107.1 mg, 18%) and fraction B (47 mg, 8%), in the form of a yellow oil. The fractions were analysed using NMR (see the detailed composition of the fractions in Table 4 and NMR data in see the Supplementary Materials, Table S1). HRMS [M + Na]^+^: *m*/*z* calcd 241.068; found 241.067.

#### 3.3.2. Synthesis of 1-*O*-Propargyl-d-Mannose (**2**) Catalysed by Sulphuric Acid

The procedure was carried out according to Roy and Mukhopadhyay [19]. Briefly: To the suspension of d-mannose 1 (0.3 g, 1.67 mmol) in propargyl alcohol (480 µL, 8.33 mmol), H_2_SO_4_⋅silica (8.8 mg) was added, and the mixture was stirred at 65 °C for 2 h. The product was then isolated by column chromatography. The excess of propargyl alcohol was first eluted with DCM, and subsequently, the product was eluted with DCM/MeOH (15:1), yielding the product 2 in the form of white solid (133.4 mg, 37%; a mixture of anomers: 26% α-2, 8% β-2, 2% α-3, and 1% β-3).

### 3.4. Synthesis Using Protective Groups

#### 3.4.1. Per-*O*-Acetyl-Mannopyranoside (**4**)

As the first step, acetylation of mannose was performed by the modified procedure described by Zhao et al. [35]. Mannose 1 (5.0 g, 27.8 mmol) was dissolved in pyridine (40 mL). Subsequently, acetic anhydride (26 mL, 275 mmol) was added dropwise at 0 °C. The mixture was slowly brought to room temperature and stirred overnight. The solution was then diluted with ethyl acetate (30 mL), extracted with 3.6% HCl (5 × 10 mL), and finally washed with brine (2 × 15 mL). Organic phases were collected, dried with anhydrous MgSO_4_, filtered, and concentrated. The crude product was purified by column chromatography (hexane/EtOAc 1:1), yielding the peracetylated product (4) in the form of a pure oil (8.32 g, 77%; a mixture of anomers: 66% α-4, 10% β-4, 1% α-5, and 0.1% β-5). HRMS [M + Na]^+^: *m/z* calcd 413.105; found 413.103; [2M + Na]^+^: *m/z* calcd 803.222; found 803.219. 

#### 3.4.2. Synthesis of Propargyl 2,3,4,6-Tetra-*O*-Acetyl-Mannopyranoside (**5**)

Acetylated propargyl mannose (**5**) was synthesised according to Poláková et al. [39]. Specifically, peracetylated mannose 4 (8.32 g, 21.3 mmol; a mixture of anomers) was dissolved in anhydrous DCM (80 mL) in an argon atmosphere. Next, propargyl alcohol (6.20 mL, 107.6 mmol) was added, and the solution was cooled to 0 °C. To the cold solution, BF_3_⋅OEt_2_ (13.2 mL, 107.2 mmol) was added dropwise; the reaction mixture was then brought to room temperature and stirred for 24 h. The solution was diluted with DCM (100 mL) and poured into ice-cold water (150 mL). The organic phase was separated, washed with saturated NaHCO_3_ (3 × 200 mL) and water (200 mL), and dried over anhydrous Na_2_SO_4_. The crude product was purified in column chromatography (hexane/EtOAc 2:1), yielding the pure product (α-6) in the form of white crystals (5.82 g, 71%). HRMS [M + Na]^+^: *m/z* calcd 409.111; found 409.109. Except for the main product (α-6), other fractions containing mixtures of anomers were isolated. The total yield of all fractions was 5.95 g. For more details on the product’s composition and analysis, see the Results and Discussion section and the Supplementary Materials, Table S1.

#### 3.4.3. Synthesis of 1-*O*-Propargyl-d-Mannose (**2**)

1-*O*-propargyl-d-mannose (**2**) was synthesised by the modified procedure described by Poláková et al. [39] and Řezanka et al. [43]. The compound α-6 (5.76 g, 14.9 mmol) was dissolved in the mixture of anhydrous DCM (10 mL) and anhydrous methanol (40 mL) in an inert atmosphere. Subsequently, sodium methoxide solution (2.6 mL) was added, and the solution was stirred under argon at room temperature for 24 h. Then, the mixture was neutralised with DOWEX H^+^ form, filtered and concentrated in vacuo. The product was purified in column chromatography (DCM/MeOH 6:1). The final product (α-2) was obtained in the form of white crystals (2.71 g, 83%). HRMS [M + Na]^+^: *m/z* calcd 241.068; found 241.067. FTIR ν: 3269 cm^−1^ (C≡C−H), 2120 cm^−1^ (C≡C).

## 4. Conclusions

Our detailed analysis of the formed products described the selectivity of d-mannose *O*^1^-propargylation. Three previously published procedures of propargyl-mannosides preparation were carried out, and the products were thoroughly examined by NMR. Our results show that one-step preparations may produce more than just the α-mannopyranoside described in the literature. Both preparations (catalysed either by hydrochloric acid or sulphuric acid on silica) resulted in the mixture of several isomers, such as α-mannopyranoside, β-mannopyranoside, α-mannofuranoside, and β-mannofuranoside. In our experience, the product mixtures cannot be separated. Therefore, the use of these preparations may be problematic in applications where the anomeric purity of the product is essential. Moreover, a three-step preparation of α-mannopyranoside was repeated. Although the mixture of anomers was produced in the first step, by-products were easily separable after the second step, and the final step resulted in pure α-mannopyranoside.

We believe that our observations are mostly general for similar alkyl groups and saccharides of similar steric hindrance around *O*^1^. We also believe that the presented data may facilitate the interpretation of NMR spectra of saccharide derivatives, which are frequently discussed in biomedical applications.

## Figures and Tables

**Table 1:** Overview of publications, in which sulphuric acid-catalysed propargylation of mannose was performed, and the yields of reactions, anomers, and their ratios are described.

Author.	Yield (%)	Described Anomers ^1^H and ^13^C NMR	Ratio of Anomers (α/β-Pyranoside)
Roy (2007) [19]	83	α-6	1:0
Shaikh (2011) [24]	90	−	1:0
Basuki (2014) [21]	30	α-6β-6	8:1 after acetylation
Su (2015) [25]	85	α-2	− (considering pure *α*-anomer)
Oz (2019) [16]	39	−	−
Raposo (2020) [20]	38	α-2	2:1

**Table 2:** Overview of papers where per-*O*-acetylation of mannose was performed. Yields of reactions, anomers, and their ratios are described.

Author.	Yield (%)	Described Anomers ^1^H and ^13^C NMR	Ratio of Anomers (α/β-Pyranoside)
Zhao (2012) [35]	95	-	Mixture with an undefined ratio
Wamhoff (2016) [36]	80	α-4 β-4	Mixture with an undefined ratio
Abellán-Flos (2016) [37]	Quant.	α-4	−

**Table 3:** Overview of preparations where propargylation of per-*O*-acetylated mannose was performed.

Author.	Yield (%)	Described Anomers ^1^H and ^13^C NMR	Ratio of Anomers (α/β-Pyranoside)
Zhao (2012) [35]	71	α-6	-
Poláková (2011) [39]	90	α-6	-
Kramer (2019) [32]	73	α-6	-
Wardrop (2002) [40]	77	α-6	1:0
Hellmuth (2017) [33]	73	α-6	-
Pohlit (2017) [41]	73	α-6	-
Spicer (2013) [42]	81	α-6	-
Wamhoff (2016) [36]	3	α-6	-
Abellán-Flos (2016) [37]	62	α-6	-

**Table 4:** The composition of fractions yielded in direct synthesis catalysed by hydrochloric acid. The composition of the combined fractions is discussed in the Results and Discussion section.

Isomer	Yield in Fraction A (%)	Yield in Fraction B (%)	Yield in Combined Fractions (%)
α-2	4.0	3.0	7.0
β-2	0.6	2.5	3.1
α-3	12.3	0.5	12.8
β-3	0.6	1.7	2.3
Total yield	17.5	7.7	25.2

## Data Availability

Data will be available upon request.

## Supplementary Materials

Complete NMR characteristics of all studied products are given in Supplementary Materials, Table S1: NMR characteristics of the studied compounds.

## Sample Availability

Samples of the compounds may be available upon request.
