# Supplementary material for: Selectivity of 1-O-Propargyl-d-Mannose Preparations
Source: Molecules. 2022 Feb 22;27(5):1483. doi: 10.3390/molecules27051483 (PMC8911549; doi:10.3390/molecules27051483)

Selectivity of 1-*O*-Propargyl-D-Mannose Preparations

Supplementary Material

Ilona Krabicová[1], Bohumil Dolenský[2] and Michal Řezanka*[3]

[1]Department of Chemistry, Faculty of Science, Humanities and Education, Technical University of Liberec, Studentská 1402/2, 461 17 Liberec, Czech Republic

[2]Department of Analytical Chemistry, Faculty of Chemical Engineering, University of Chemistry and Technology Prague, Technická 5, 166 28 Prague, Czech Republic

[3]Department of Nanochemistry, Institute for Nanomaterials, Advanced Technologies and Innovation, Technical University of Liberec, Studentská 1402/2, 461 17 Liberec, Czech Republic

The characteristics of each compound in the following table were usually derived based on combine analyses of several 1D and 2D NMR spectra of samples of various composition. That include not only common 1D, but also 2D spectra (COSY, DQF-COSY, LR-COSY, TOCSY, NOESY, ROESY, HSQC, HSQC-NOESY, HSQC-TOCSY, HMBC, HSQMBC) to confirm the molecular structures and, but also to uncover and solve the overlaid signals. The series of selective 1D NOESY, ROESY, and TOCSY spectra, and the spectra with selective homodecoupling were crucial as well. Since most of the samples contain compounds of very different contents (see the manuscript) there is not a proper plotting scale for the spectra. Hence we show only selected spectra of all observed compounds, which also demonstrate the complexity and the ways of our analyses.

NMR spectral data

Table S1. NMR characteristics of the studied compounds

Compound	C1<br>H1	C2<br>H2	C3<br>H3	C4<br>H4	C5<br>H5	C6<br>H6
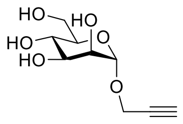<br>***α*-2**<br>in CD_3OD	99.819<br>4.964 dt<br>J_{HH} = 1.7, 0.5<br>$^1J_{HC}$ = 169.6<br><br>**O1-CH_2C≡CH**<br>54.808<br>4.273 d<br>J_{HH} = 2.4<br>$^1J_{HC}$ = 149.3<br>75.959<br>80.018<br>2.850 t<br>J_{HH} = 2.4<br>$^1J_{HC}$ = 251.0<br>$^2J_{HC}$ = 49.9	71.997<br>3.795 dd<br>J_{HH} = 3.3, 1.7<br><br>remaining OH<br>5.461 very br d	72.473<br>3.670 ddd<br>J_{HH} = 9.4, 3.3, 0.4<br><br>remaining OH<br>5.216 very br d	68.455<br>3.620 t<br>J_{HH} = 9.5<br><br>remaining OH<br>5.412 very br d	75.063<br>3.509 dddd<br>J_{HH} = 9.6, 5.9, 2.4, 0.6	62.807<br>3.835 dd<br>J_{HH} = 11.8, 2.4<br>3.704 dd<br>J_{HH} = 11.8, 5.9<br><br>remaining OH<br>5.242 very br t

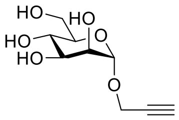<br>***α*-2**<br>in D_2O	98.798<br>4.927 dm<br>J_{HH} = 1.8<br>**O1-CH_2C≡C-H (or -D)**<br>54.599<br>(54.590) [a]<br>4.250 dd<br>J_{HH} = 15.9, 2.4<br>4.206 dd<br>J_{HH} = 15.9, 2.4<br>78.873<br>(78.422, t, J_{CD} 7.7) [a]<br>76.212<br>(75.986, t, J_{CD} 39.2) [a]<br>2.817 t<br>J_{HH} = 2.5	69.977<br>3.846 ddd<br>J_{HH} = 3.5, 1.8, 0.3	70.500<br>3.689 ddd<br>J_{HH} = 9.5, 3.5, 0.4	66.663<br>3.575 ~t [b]<br>J_{HH} = ~9.8	73.173<br>~3.563 m [b]	60.858<br>3.782 dd<br>J_{HH} = 12.3, 1.9, 0.4<br>3.663 dd<br>J_{HH} = 12.3, 5.6
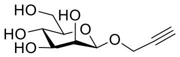<br>***β*-2**<br>in D_2O	98.104<br>4.757 d<br>J_{HH} = 1.0	70.500<br>3.901 ddd<br>J_{HH} = 3.3, 1.0, 0.3	72.961<br>3.563 dd<br>J_{HH} = 9.8, 3.3	66.882<br>3.478 t<br>J_{HH} = 9.7	76.406<br>3.293 ddd<br>J_{HH} = 9.8, 6.5, 2.3	61.098<br>3.829 dd<br>J_{HH} = 12.3, 2.3, 0.3<br>3.633 d<br>J_{HH} = 12.3, 6.5

	**O1-$CH_2C{\equiv}CH$**<br>56.070<br>4.364 dm [b]<br>J_{HH} = 16.0<br>4.348 dm [b]<br>J_{HH} = 16.0<br>78.968<br>76.277<br>2.820 t<br>J_{HH} = 2.4					
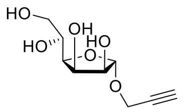<br>***α*-3**<br>in D_2O	100.235<br>5.081 dd<br>J_{HH} = 4.9, 0.4<br><br>**O1-$CH_2C{\equiv}CH$**<br>55.400<br>4.213 d<br>J_{HH} = 2.4<br>79.391<br>75.837<br>2.780 t<br>J_{HH} = 2.4	71.899<br>4.157 dd<br>J_{HH} = 5.1, 4.9	69.659<br>4.216 dd<br>J_{HH} = 5.1, 4.1	79.840<br>3.926 ddd<br>J_{HH} = 9.3, 4.1, 0.4	69.867<br>3.880 ddd<br>J_{HH} = 9.3, 5.8, 2.6	63.049<br>3.747 dd<br>J_{HH} = 12.0, 2.6<br>3.586 dd<br>J_{HH} = 12.0, 5.8

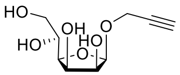<br>***β*-3**<br>in D_2O	106.488<br>5.080 ddd<br>J_{HH} = 4.1, 0.4, 0.3<br>**O1-$CH_2C≡C$-H (or -D)**<br>55.880<br>(55.872) [a]<br>4.260 dd<br>J_{HH} = 15.9, 2.4<br>4.218 dd<br>J_{HH} = 15.9, 2.4<br>79.092<br>(78.642, t, J_{CD} 7.5) [a]<br>76.100<br>(75.870, t, J_{CD} 38.6) [a]<br>2.808 t<br>J_{HH} = 2.5	76.967<br>4.099 dd<br>J_{HH} = 4.7, 4.1	71.234<br>4.241 dddd<br>J_{HH} = 4.7, 3.0, 0.4, 0.2	79.579<br>3.976 dd<br>J_{HH} = 8.8, 3.0, 0.3	69.028<br>3.840 ddd<br>J_{HH} = 8.8, 6.1, 2.9	63.075<br>3.708 dd<br>J_{HH} = 12.1, 2.9, 0.2<br>3.533 dd<br>J_{HH} = 12.1, 6.1
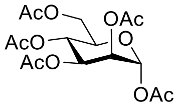<br>***α*-4**<br>in $CDCl_3$	90.690<br>6.058 dd<br>J_{HH} = 2.0, 0.7<br>J_{HC} = 177.8	68.422<br>5.234 m [b]	68.825<br>~5.32 m [b]	65.630<br>~5.32 m [b]	70.693<br>~4.026 m [b]	62.186<br>4.253 dd<br>J_{HH} = 12.4, 4.9<br>4.077 dd<br>J_{HH} = 12.4, 2.5

	**O1-Ac**<br>168.148<br>20.919<br>2.144 s	**O2-Ac**<br>169.821<br>20.824<br>2.151 s	**O3-Ac**<br>170.071<br>20.696<br>1.981 s	**O4-Ac**<br>169.621<br>20.718<br>2.028 s		**O6-Ac**<br>170.722<br>20.770<br>2.067 s
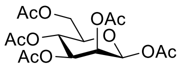<br>***β*-4**<br>in $CDCl_3$	90.509<br>5.840 d<br>J_{HH} = 1.2<br><br>**O1-Ac**<br>168.456<br>20.784<br>2.076 s	68.273<br>5.458 ddd<br>J_{HH} = 3.3, 1.2, 0.4<br><br>**O2-Ac**<br>170.282<br>20.830<br>2.187 s	70.728<br>5.112 dd<br>J_{HH} = 10.0, 3.3<br><br>**O3-Ac**<br>169.875<br>20.597<br>1.978 s	65.505<br>5.263 td<br>J_{HH} = 9.9, 0.4<br><br>**O4-Ac**<br>169.665<br>20.734<br>2.028 s	73.368<br>3.786 ddd<br>J = 9.9, 5.4, 2.4	62.151<br>4.279 dd<br>J_{HH} = 12.4, 5.4<br>4.116 dd<br>J_{HH} = 12.4, 2.4<br><br>**O6-Ac**<br>170.736<br>20.807<br>2.067 s
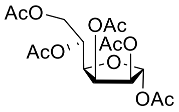<br>***α*-5**<br>in $CDCl_3$	98.48<br>6.206 ddd<br>J_{HH} = 3.4, 0.5, 0.4	75.45<br>5.35 dd<br>J_{HH} = 5.0, 3.4	70.39<br>5.59 ddd<br>J_{HH} = 5.0, 4.2, 0.4	77.44<br>4.46 dd<br>J_{HH} = 8.7, 4.2	signal is covered<br>5.27<br>J_{HH} = 8.7, 5.8, 2.4	62.69<br>4.56 dd<br>J_{HH} = 12.3, 2.5<br>4.07 dd<br>J_{HH} = 12.3, 5.8
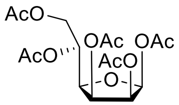<br>***β*-5**<br>in $CDCl_3$	6.32 dd<br>J_{HH} = 4.8, 0.5	5.24 ~t [b]<br>J_{HH} ~ 5.0	5.62 dd<br>J_{HH} = 5.4, 4.8	4.39 dd<br>J_{HH} = 9.4, 4.7	not observed due to overlays and low content	not observed due to overlays and low content

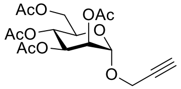<br>***α*-6**<br>in C_6D_6	96.828<br>4.954 ddd<br>J_{HH} = 1.8, 0.7, 0.4<br>**O1-CH_2C≡CH**<br>54.837<br>3.818 dd<br>J_{HH} = 15.8, 2.4<br>3.767 dd<br>J_{HH} = 15.8, 2.5<br>78.385<br>75.666<br>1.925 t<br>J_{HH} = 2.4	69.953<br>5.566 dd<br>J_{HH} = 3.2, 1.8<br>**O2-Ac**<br>169.534<br>20.146<br>1.584 s	69.639<br>5.658 dd [b]<br>J_{HH} = 10.2, 3.2<br>**O3-Ac**<br>169.461<br>20.293<br>1.696 s	66.308<br>5.703 dd [b]<br>J_{HH} = 10.2, 9.5<br>**O4-Ac**<br>169.392<br>20.218<br>1.654 s	69.753<br>3.899 dddd<br>J_{HH} = 9.5, 4.8, 2.5, 0.7	62.301<br>4.361 dd<br>J_{HH} = 12.3, 4.8<br>4.128 dd<br>J_{HH} = 12.3, 2.5<br>**O6-Ac**<br>170.030<br>20.321<br>1.722 s
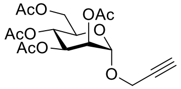<br>***α*-6**<br>in $CDCl_3$	96.363<br>5.014 ddd<br>J_{HH} = 1.8, 0.7, 0.4	69.466<br>5.255 dd<br>J_{HH} = 3.6, 1.8	69.033<br>5.32 covered	66.143<br>5.28 dd<br>J_{HH} = 10.1, 9.5	69.102<br>4.005 dddd<br>J_{HH} = 9.8, 5.2, 2.5, 0.7	62.433<br>4.269 dd<br>J_{HH} = 12.3, 5.2<br>4.096 dd<br>J_{HH} = 12.3, 2.5

	**O1-$CH_2C{\equiv}CH$**<br>55.064<br>4.295 d<br>J_{HH} = 2.4<br>75.706<br>78.035<br>2.465 t<br>J_{HH} = 2.4<br>$^1J_{HC}$ = 251.8<br>$^2J_{HC}$ = 49.6	**O2-Ac**<br>170.047<br>20.957<br>2.145 s	**O3-Ac**<br>169.941<br>20.748<br>1.973 s	**O4-Ac**<br>169.798<br>20.781<br>2.023 s		**O6-Ac**<br>170.736<br>20.836<br>2.086 s
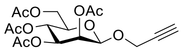<br>***β*-6**<br>in $CDCl_3$	95.752<br>4.936 d<br>J_{HH} = 1.2<br><br>**O1-$CH_2C{\equiv}CH$**<br>55.896<br>4.35 dd<br>J_{HH} = 16.0, 2.5<br>3.39 dd<br>J_{HH} = 16.0, 2.4<br>77.931<br>76.715<br>2.481 t<br>J_{HH} = 2.4	68.842<br>5.472 dd<br>J_{HH} = 3.3, 1.2<br><br>**O2-Ac**<br>170.396<br>20.926<br>2.168 s	71.199<br>5.081 dd<br>J_{HH} = 10.0, 3.3<br><br>**O3-Ac**<br>170.134<br>20.672<br>1.979 s	66.053<br>5.253 t<br>J_{HH} = 10.0<br><br>**O4-Ac**<br>169.705<br>20.788<br>2.030 s	72.637<br>3.690 ddd<br>J_{HH} = 9.9, 5.3, 2.6	62.432<br>4.301 dd<br>J_{HH} = 12.3, 5.3<br>4.145 dd<br>J_{HH} = 12.3, 2.6<br><br>**O6-Ac**<br>170.824<br>20.854<br>2.081 s

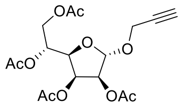<br>***α*-7**<br>in $CDCl_3$	103.559<br>5.300 dt<br>J_{HH} = 2.8, 0.5<br>**O1-CH_2C≡CH**<br>55.356<br>2.27 dd<br>J_{HH} = 15.7, 2.5<br>4.22 dd<br>J_{HH} = 15.7, 2.4<br>78.547<br>75.209<br>2.455 t<br>J_{HH} = 2.4	76.458<br>5.230 dd<br>J_{HH} = 5.2, 2.8<br>**O2-Ac**<br>169.432<br>20.509<br>2.066 s	70.760<br>5.586 ddd<br>J_{HH} = 5.2, 4.3, 0.5<br>**O3-Ac**<br>169.597<br>20.471<br>2.047 s	76.289<br>4.380 ddd<br>J_{HH} = 8.9, 4.3, 0.5	68.177<br>5.279 ddd<br>J_{HH} = 8.9, 5.6, 2.4<br>**O5-Ac**<br>169.822<br>20.900<br>2.006 s	63.003<br>4.544 dd<br>J_{HH} = 12.3, 2.4<br>4.147 dd<br>J_{HH} = 12.3, 2.6<br>**O6-Ac**<br>170.785<br>20.866<br>2.062 s
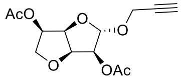<br>***α*-8**<br>in $CDCl_3$	105.541<br>5.372 dq<br>J_{HH} = 1.2, 0.5	77.673<br>5.048 dd<br>J_{HH} = 6.1, 1.2	80.360<br>4.848 ddd<br>J_{HH} = 6.1, 5.0, 0.6	79.324<br>4.793 br t<br>J_{HH} = 5.1	72.578<br>4.990 dddd<br>J_{HH} = 9.1, 6.9, 5.1, 0.6	69.104<br>3.685 ddd<br>J_{HH} = 9.1, 8.5, 0.4<br>4.087 dd<br>J_{HH} = 8.5, 6.9

	**O1-$CH_2C≡CH$**	**O2-Ac**			**O5-Ac**	
	54.575	169.798			170.447	
	2.24 dd	20.555			20.794	
	J_{HH} = 15.7, 2.4	2.120 s			2.137 s	
	4.20 dd					
	J_{HH} = 15.7, 2.4					
	not observed					
	75.131					
	2.445 t					
	J_{HH} = 2.4					

[a] The signal for the isotopologue having deuterium on the propargyl group, i.e., O1-CH_2-C≡CD

[b] The signal suffers from second order effects

Spectrum 1. The ^{1}H NMR spectra of the samples having various contents of pyranosides *α*-**2** (green) and *β*-**2** (blue), and furanosides *α*-**3** (violet) and *β*-**3** (red) in D_2O.

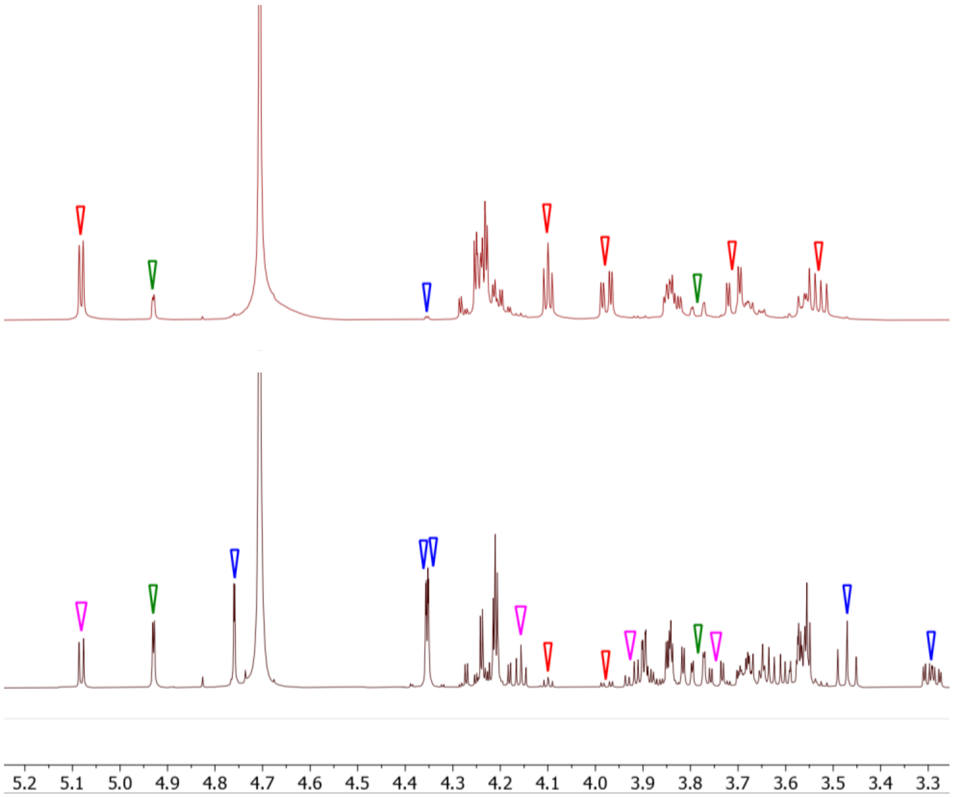

Spectrum 2. The 1D NOESY and ROESY experiments (mixing times 200, 400 and 800 ms) on the signal of H1 hydrogen of *β*-**2** within the mixture of pyranosides *α*-**2** and *β*-**2**, and furanosides *α*-**3** and *β*-**3** in D_2O; the bottom spectrum is the ^{1}H NMR spectrum with suppressing of the water signal via DANTE.

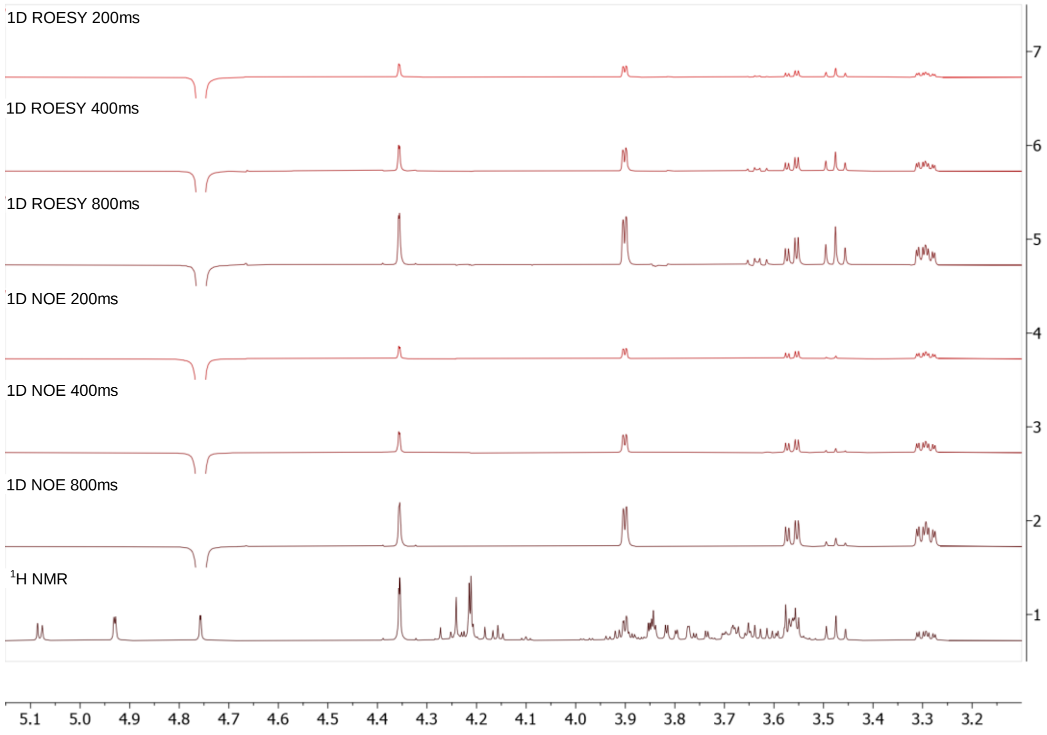

Spectrum 3. The 1D TOCSY experiments (mixing times 40, 60, 100, 200 and 300 ms) on the signal of H1 hydrogen of ***β*-3** within the mixture of pyranosides ***α*-2** and ***β*-2**, and furanosides ***α*-3** and ***β*-3** in D_2O; the bottom spectrum is the ordinary ^{1}H NMR spectrum.

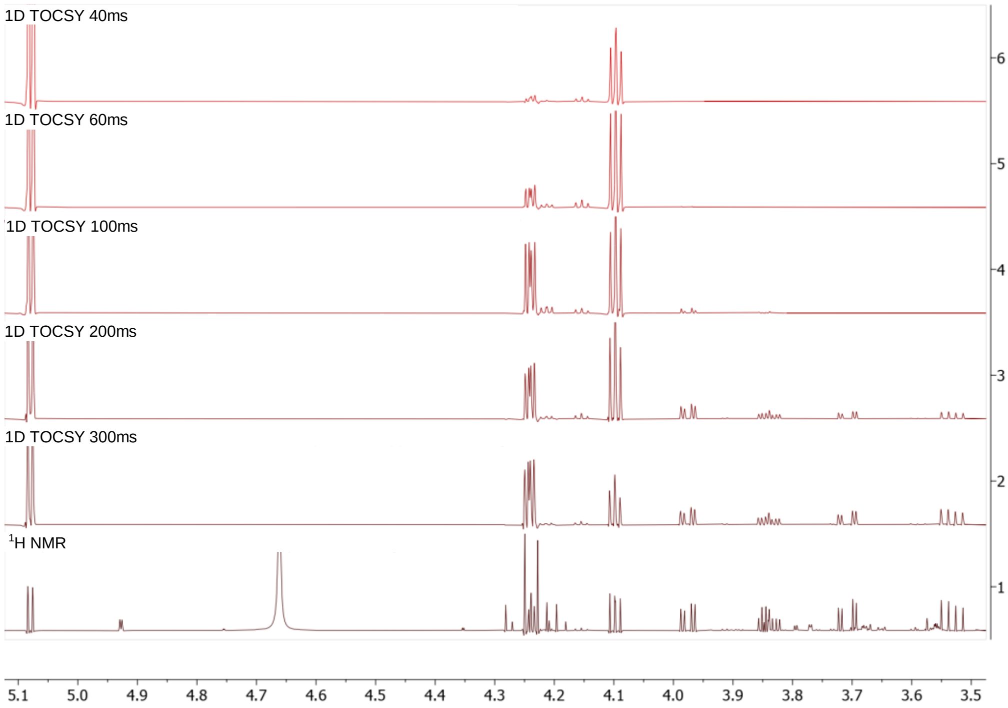

Spectrum 4. The ^{13}C NMR spectra of the samples prepared by HCl-catalysed one-step synthesis having various contents of pyranosides ***α*-2** (green) and ***β*-2** (blue), and furanosides ***α*-3** (violet) and ***β*-3** (red) in D_2O.

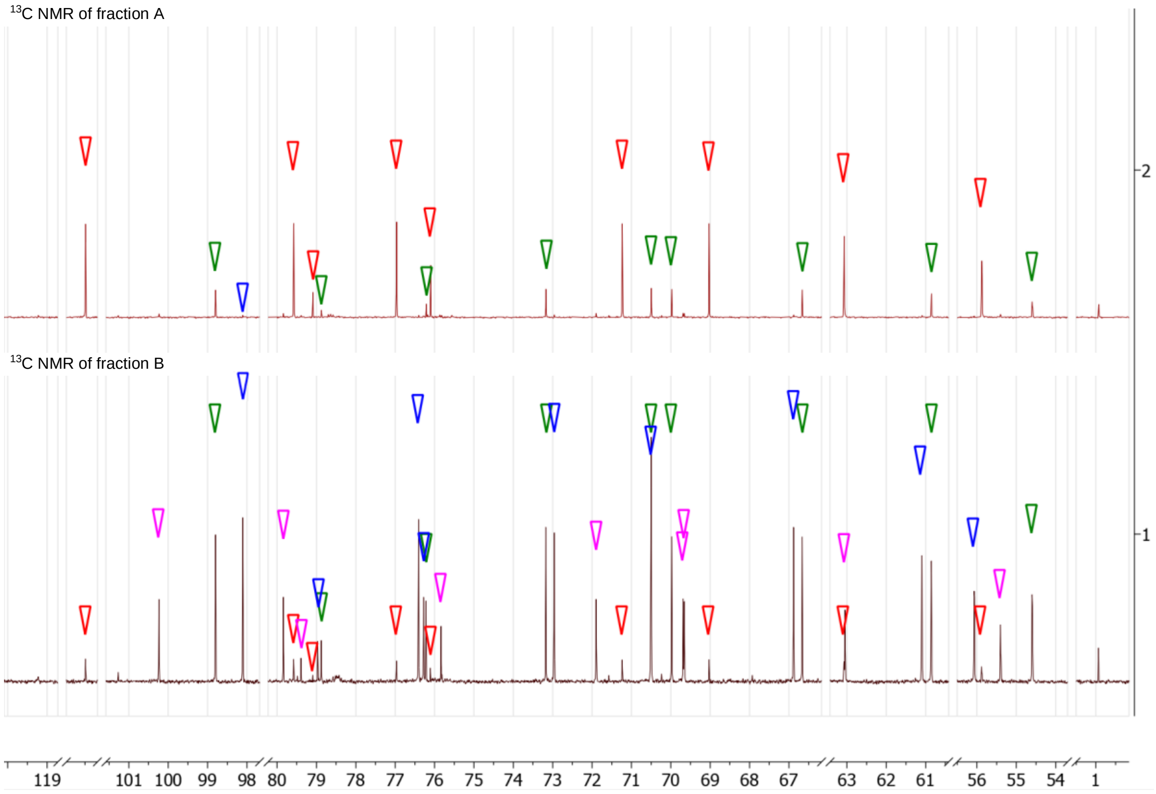

Spectrum 5. The 2D HSQC-NOESY spectrum on of pyranosides α-**2** and β-**2**, and furanosides α-**3** and β-**3** in D_2O.

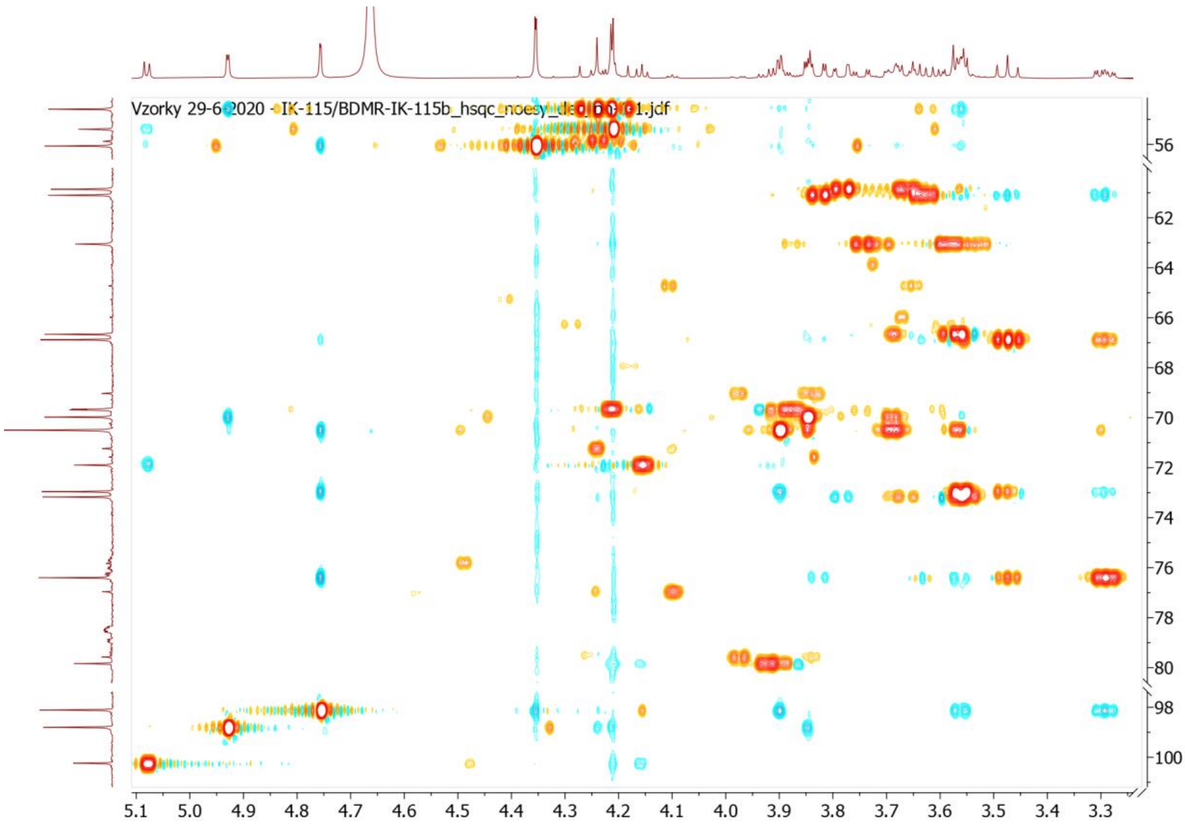

Spectrum 6. The ^{13}C APT NMR spectrum of pyranosides α-**4** (major) and β-**4** (minor) mixture in $CDCl_3$.

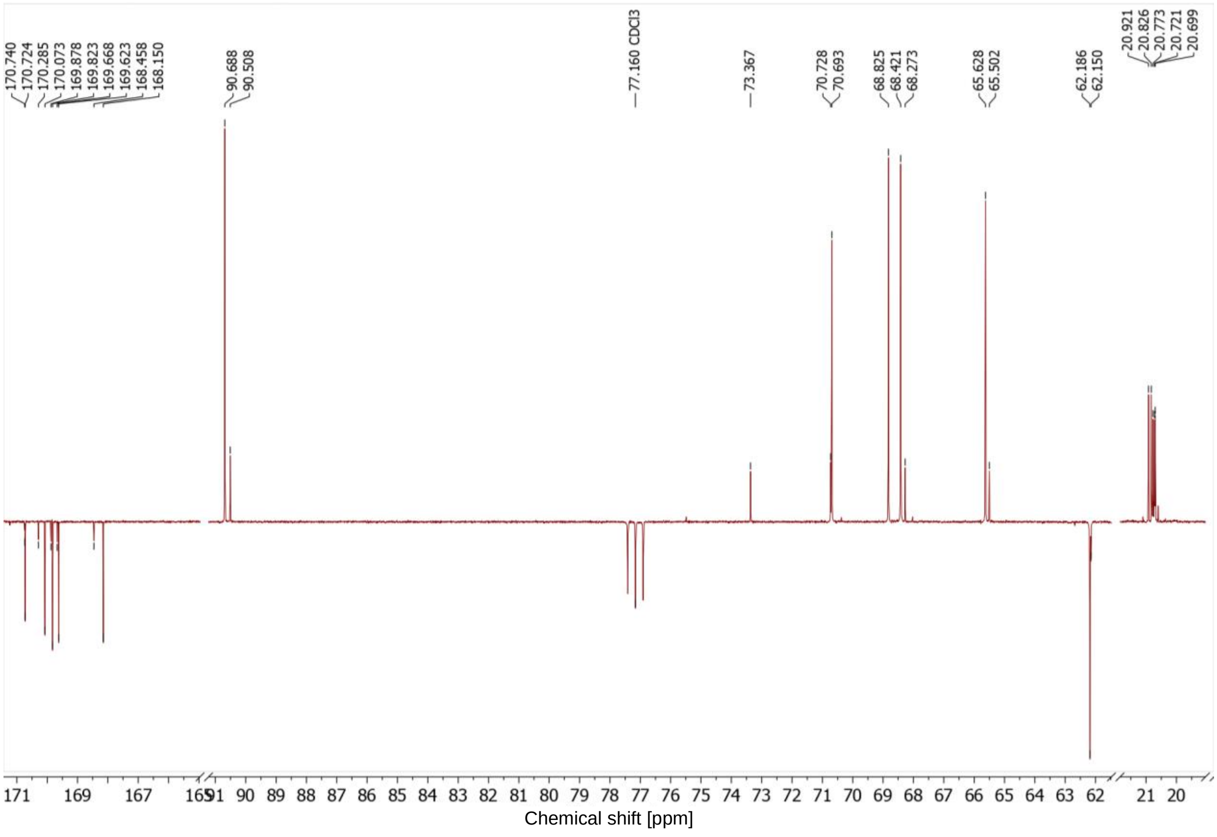

Spectrum 7. The ^{1}H NMR spectrum of pyranosides *α*-**4** and *β*-**4** with traces of furanosides *α*-**5** and *β*-**5** in $CDCl_3$.

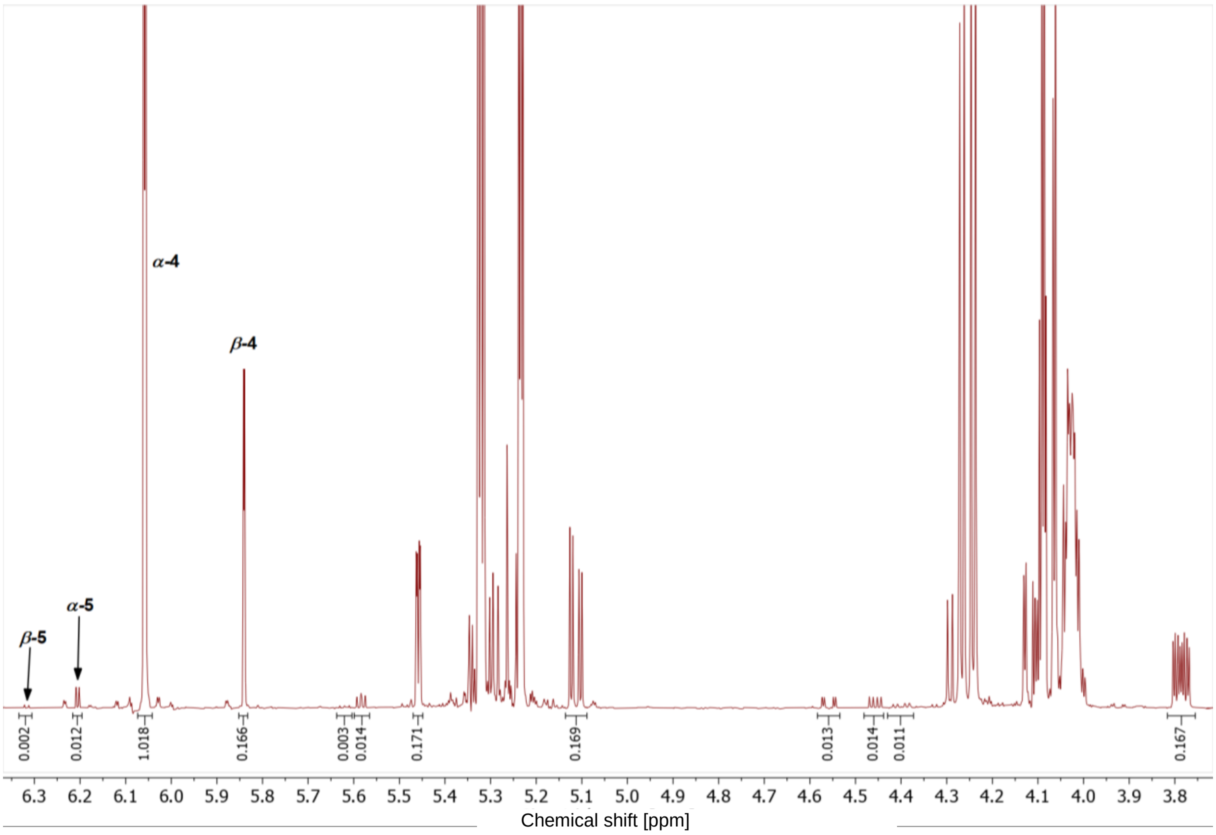

Spectrum 8. The ^{1}H NMR spectrum of pyranoside *α*-**6** in C_6D_6.

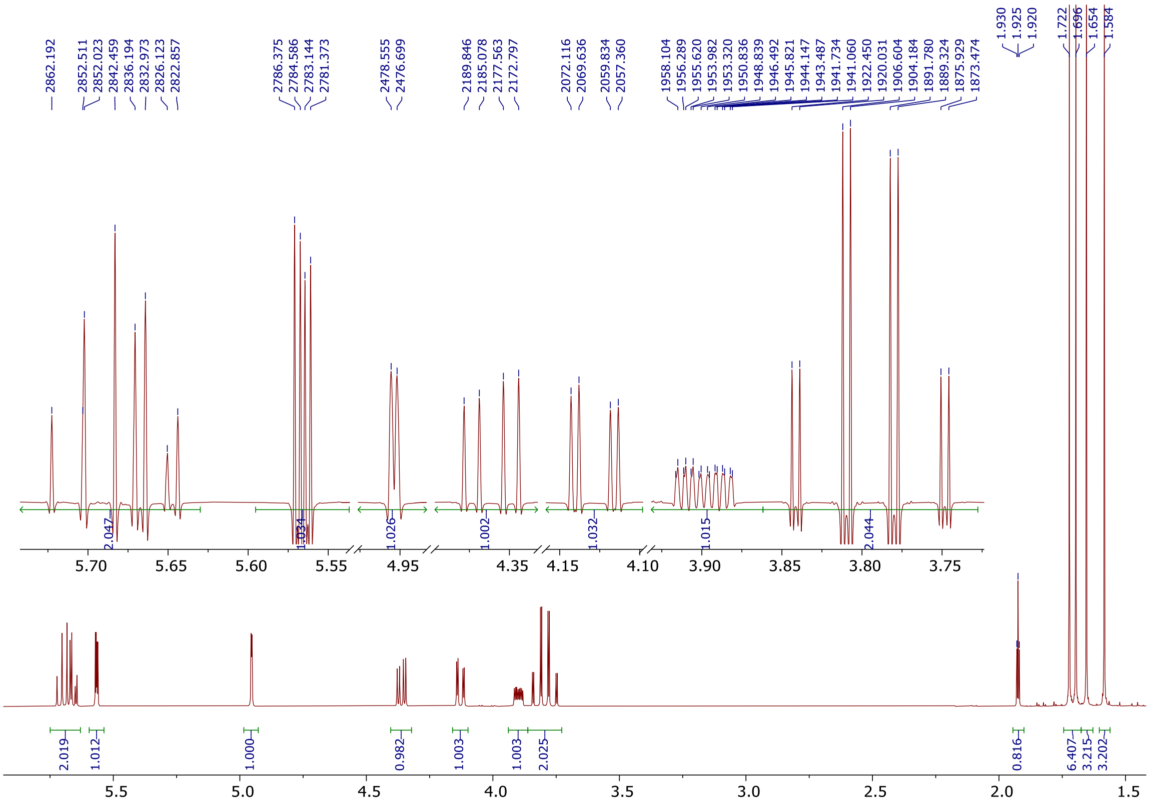

Spectrum 9. The ^{13}C APT NMR spectrum of pyranoside α**-6** in C_6D_6.

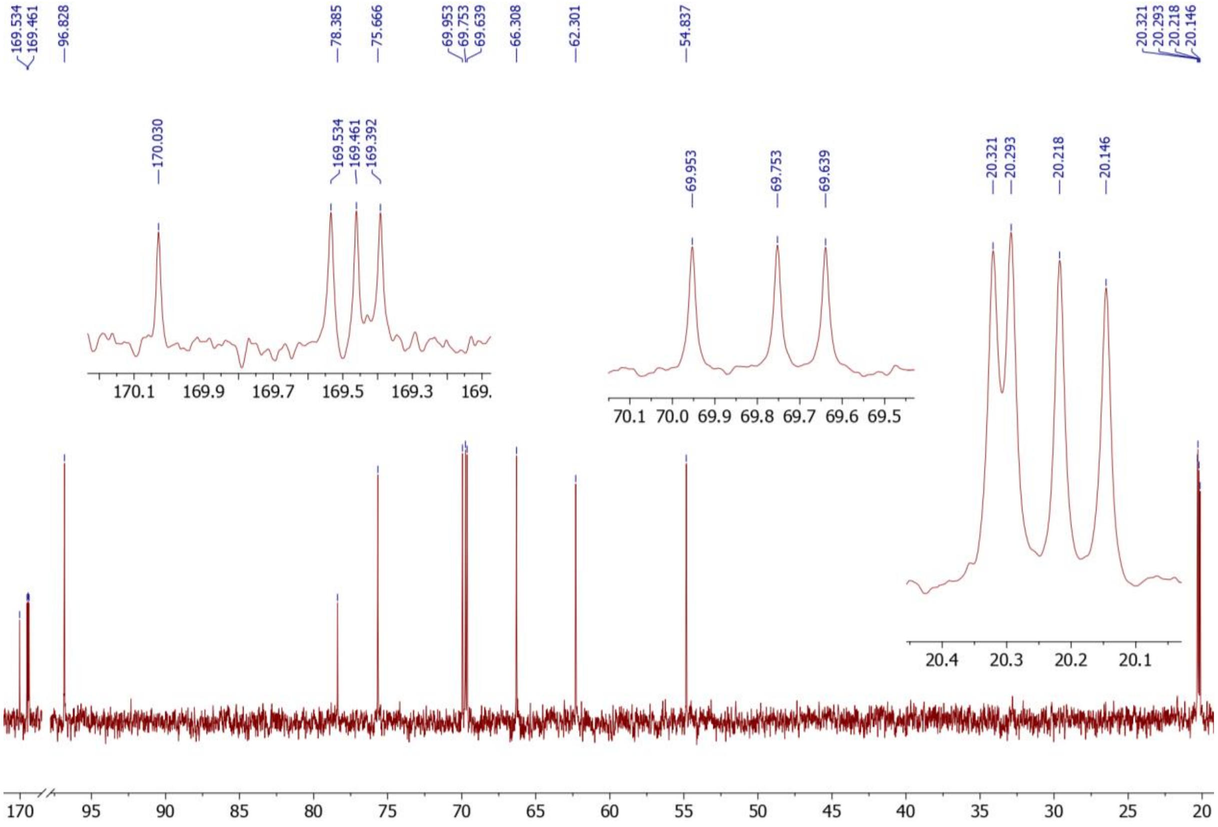

Spectrum 10. The ^{1}H NMR spectra of pyranosides α**-7** (major) and anhydromannose α**-8** (minor) in $CDCl_3$.

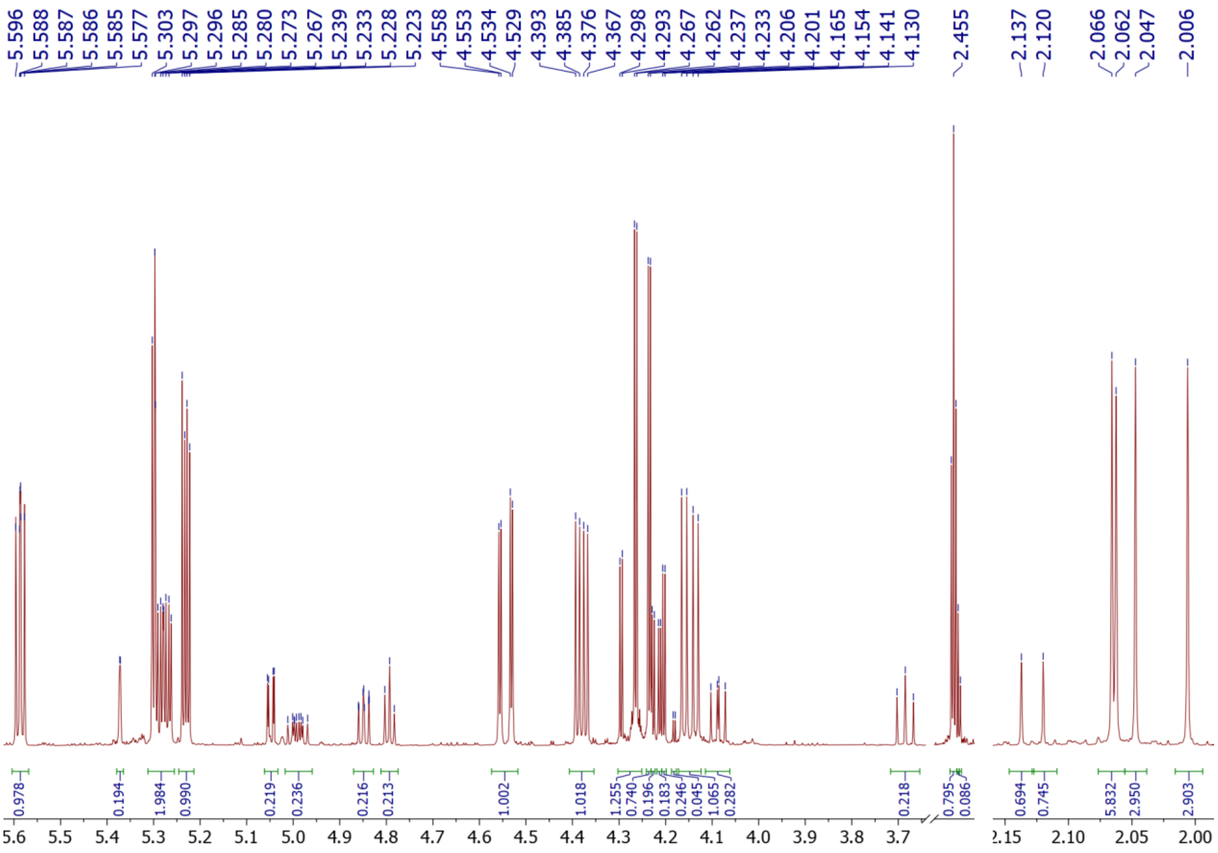

Spectrum 11. The ^{13}C APT NMR spectra of pyranosides *α*-**7** (major) and anhydromannose *α*-**8** (minor) in $CDCl_3$.

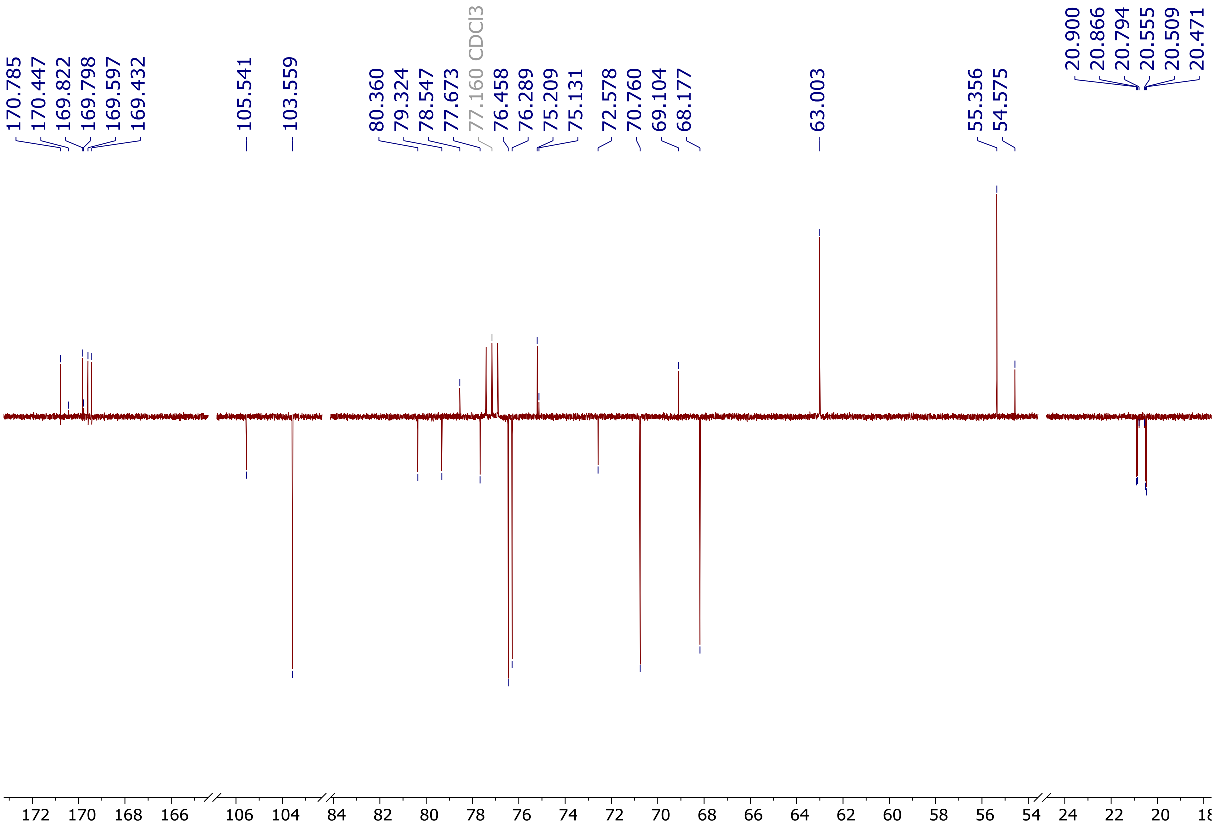

Supplement: Supplementary file 1 [file molecules-27-01483-s001.zip › molecules-1594953-supplementary.pdf]
